# Supplementary material for: Gastrointestinal microbiota composition predicts peripheral inflammatory state during treatment of human tuberculosis
Source: Nat Commun. 2021 Feb 18;12:1141. doi: 10.1038/s41467-021-21475-y (PMC7892575; doi:10.1038/s41467-021-21475-y)
Supplement: Supplementary file 2 — Description of Additional Supplementary Files [file 41467_2021_21475_MOESM2_ESM.pdf]

Description of Additional Supplementary Files

File Name: Supplementary Data 1
Description: Table reporting first and late date of enrolment for each study Cohort.

File Name: Supplementary Data 2
Description: Results from Linear Mixed Effect Modelling predicting TTP for the HRZE/NTZ Cohort.

File Name: Supplementary Data 3
Description: Results from Linear Mixed Effect Modelling predicting microbiome Diversity for the HRZE/NTZ Cohort.

File Name: Supplementary Data 4
Description: Results PERMANOVA analysis on microbiota abundance for the HRZE/NTZ study Cohort.

File Name: Supplementary Data 5
Description: Results from differential analysis using limma/voom to determine bacteria that are affected by NTZ and HRZE treatment.

File Name: Supplementary Data 6
Description: MiSigDB Hallmark pathways affected by NTZ and HRZE treatment for the HRZE/NTZ study Cohort.

File Name: Supplementary Data 7
Description: TB inflammatory transcripts that are affected by HRZE for the HRZE/NTZ study Cohort.

File Name: Supplementary Data 8
Description: TB inflammatory transcripts that are affected by NTZ for the HRZE/NTZ study Cohort.

File Name: Supplementary Data 9
Description: IBD-associated transcripts that are affected by HRZE for the HRZE/NTZ study Cohort.

File Name: Supplementary Data 10
Description: IBD-associated transcripts that are affected by NTZ for the HRZE/NTZ study Cohort.

File Name: Supplementary Data 11
Description: Results from Linear Mixed Effect Modelling predicting TTP for the Longitudinal Observational Cohort.

File Name: Supplementary Data 12
Description: Results from Linear Mixed Effect Modelling predicting microbiota Diversity for the Longitudinal Observational Cohort.

File Name: Supplementary Data 13
Description: Results from differential analysis using limma/voom to determine bacteria that are affected by HRZE treatment for the Longitudinal Observational Cohort.

File Name: Supplementary Data 14
Description: Results from differential analysis using limma/voom to determine host transcripts that are affected by HRZE treatment for the Longitudinal Observational Cohort.

File Name: Supplementary Data 15
Description: MiSigDB Hallmark pathways that are affected by HRZE for the Longitudinal Observational Cohort.

File Name: Supplementary Data 16
Description: TB inflammatory transcripts that are affected by HRZE for the Longitudinal Observational Cohort.

File Name: Supplementary Data 17
Description: IBD inflammatory transcripts that are affected by HRZE for the Longitudinal Observational Cohort.

File Name: Supplementary Data 18
Description: Results from Random Forest Regression analysis to predict changes in inflammatory pathways from changes in microbiota.

File Name: Supplementary Data 19
Description: Results from linear mixed effect modelling to regress the Euclidean distance between every pair of FC/CC and pre-treatment or during treatment samples against Time, Treatment and patient ID for the HRZE/NTZ trial.

File Name: Supplementary Data 20
Description: Results from linear mixed effect modelling to regress the Euclidean distance between every pair of FC/CC and pre-treatment or during treatment samples against Time, Treatment and patient ID for the Longitudinal Observational Cohort.

File Name: Supplementary Data 21
Description: Results from Random Forest Regression analysis to predict changes inflammatory pathways from changes microbiota abundance in the Family Contact/Community Control Cohort.
