# Supplementary material for: Gastrointestinal microbiota composition predicts peripheral inflammatory state during treatment of human tuberculosis
Source: Nat Commun. 2021 Feb 18;12:1141. doi: 10.1038/s41467-021-21475-y (PMC7892575; doi:10.1038/s41467-021-21475-y)
Supplement: Supplementary file 24 — Reporting Summary [file 41467_2021_21475_MOESM24_ESM.pdf]

nature research

Corresponding author(s): Vanni Bucci & Michael Glickman

Last updated by author(s): 22 Oct 2020 (revised Jan 2020 post acceptance

Reporting Summary

Nature Research wishes to improve the reproducibility of the work that we publish. This form provides structure for consistency and transparency in reporting. For further information on Nature Research policies, see our Editorial Policies and the Editorial Policy Checklist.

Statistics

For all statistical analyses, confirm that the following items are present in the figure legend, table legend, main text, or Methods section.

n/a	Confirmed	
☐	☒	The exact sample size (*n*) for each experimental group/condition, given as a discrete number and unit of measurement
☐	☒	A statement on whether measurements were taken from distinct samples or whether the same sample was measured repeatedly
☐	☒	The statistical test(s) used AND whether they are one- or two-sided<br>*Only common tests should be described solely by name; describe more complex techniques in the Methods section.*
☐	☒	A description of all covariates tested
☐	☒	A description of any assumptions or corrections, such as tests of normality and adjustment for multiple comparisons
☐	☒	A full description of the statistical parameters including central tendency (e.g. means) or other basic estimates (e.g. regression coefficient) AND variation (e.g. standard deviation) or associated estimates of uncertainty (e.g. confidence intervals)
☐	☒	For null hypothesis testing, the test statistic (e.g. *F*, *t*, *r*) with confidence intervals, effect sizes, degrees of freedom and *P* value noted<br>*Give P values as exact values whenever suitable.*
☐	☒	For Bayesian analysis, information on the choice of priors and Markov chain Monte Carlo settings
☐	☒	For hierarchical and complex designs, identification of the appropriate level for tests and full reporting of outcomes
☒	☐	Estimates of effect sizes (e.g. Cohen's *d*, Pearson's *r*), indicating how they were calculated

Our web collection on statistics for biologists contains articles on many of the points above.

Software and code

Policy information about availability of computer code

Data collection	No code was used for data collection.

Data analysis

Forward and reverse 16S MiSeq-generated amplicon sequencing reads were dereplicated and sequences were inferred using dada2.46 Potentially chimeric sequences were removed using consensus-based methods. Taxonomic assignments were made using BLASTN against the NCBI refseq rna database. These files were imported into R and merged with a metadata file into a single Phyloseq object. Host RNAseq sequences were aligned to the human genome (version hg38) using STAR aligner and transcript counts were estimated using featurecounts. Quality of aligned and counted reads was assessed using Quality of RNA-Seq ToolSet (QoRTs). Deseq2 in R was used to normalize sequencing counts from both 16 rRNA sequencing and peripheral blood host RNAseq. Limma and edgeR were used to analyze linear mixed effect modeling RNAseq data. PERMANOVA was performed using the Adonis function in the Vegan R package. R packages RandomForest, Boruta and Ranger were used to perform Random Forest Regression and selection of statistically significant microbes affecting host gene expression. ssGSEA (single sample gene set enrichment analysis) method was used to profile within-sample differences between pathways from the MiSigDB Hallmark pathways list with the GSVA package in R. Code to analyze the data (including 16S rRNA informatics analysis) and to reproduce all the figures and results is available on Github at https://wipperman.github.io/TBRU/. R version 3.6 was used.

All analysis statistical and computational analysis was performed in R v.3.6.1 (2019-07-05) with Platform: x86_64-pc-linux-gnu (64-bit), running under: Ubuntu 16.04.6 LTS. The following R packages were used: nlme (v.3.1-141), reshape2 (v.1.4.4), ALEPlot (v.1.1), vita (v.1.0.0), randomForest (v.4.6-14), GSVA (v.1.32.0) msigdbr (v.7.1.1), fgsea (v.1.10.1), Rcpp (v.1.0.5), forcats (v.0.5.0), purrr (v.0.3.4), readr (v.1.3.1), tidyr (v.1.1.2), tibble (v.3.0.4), tidyverse (v.1.3.0), RColorBrewer (v.1.1-2), ggthemes (v.4.2.0), hues (v.0.2.0.9000), edgeR (v.3.26.8), variancePartition (v.1.14.1), scales (v.1.1.1), foreach (v.1.5.1), limma (v.3.40.6), circlize (v.0.4.10), ComplexHeatmap (v.2.0.0), gtools (v.3.8.2), ggplot2 (v.3.3.2), stringr (v.1.4.0), ifultools (v.2.0-5), data.table (v.1.13.0), yingtools2 (v.0.0.0.62), dplyr (v.1.0.2), phyloseq (v.1.28.0), DESeq2 (v.1.24.0), SummarizedExperiment (v.1.14.1), DelayedArray (v.0.10.0), BiocParallel (v.1.18.1), matrixStats (v.0.56.0), Biobase (v.2.44.0), GenomicRanges (v.1.36.1), GenomeInfoDb (v.1.20.0), IRanges (v.2.18.3), S4Vectors (v.0.22.1), BiocGenerics (v.0.30.0).

For manuscripts utilizing custom algorithms or software that are central to the research but not yet described in published literature, software must be made available to editors and reviewers. We strongly encourage code deposition in a community repository (e.g. GitHub). See the Nature Research guidelines for submitting code & software for further information.

Data

Policy information about availability of data

All manuscripts must include a data availability statement. This statement should provide the following information, where applicable:

- Accession codes, unique identifiers, or web links for publicly available datasets
- A list of figures that have associated raw data
- A description of any restrictions on data availability

Data on Time to Positivity where obtained from Walsh et al. 2020 and are available on Github at https://wipperman.github.io/TBRU/. 16S rDNA sequencing data is deposited with the SRA under accession no. PRJNA445968. Peripheral blood transcriptomic data are deposited with the SRA under accession no. PRJNA445968.

Field-specific reporting

Please select the one below that is the best fit for your research. If you are not sure, read the appropriate sections before making your selection.

☒ Life sciences ☐ Behavioural & social sciences ☐ Ecological, evolutionary & environmental sciences

For a reference copy of the document with all sections, see nature.com/documents/nr-reporting-summary-flat.pdf

Life sciences study design

All studies must disclose on these points even when the disclosure is negative.

Sample size

No formal sample size considerations were used in this analysis. This study was not powered for RNAseq data analysis, rather, it was powered for the primary endpoint--Time to Positivity (TTP) in Mtb.

This study is powered to answer the scientific questions addressed in this paper. The fold changes we are powered to detect with RNAseq data using the following assumptions are reasonable and consistent with other RNAseq studies.

We ran power calculations to determine that to determine that with 16 pre and 16 post treatment microbiome samples and 8 pre and 8 post treatment RNAseq samples for the HRZE cohort, with 80% power at significance level of 0.05, we could detect a fold change of 1.4 for microbiome difference and a fold change of 1.8 for mRNA transcripts. In the NTZ cohort, with 18 pre and 18 post treatment microbiome samples and 14 pre and 14 post treatment RNAseq samples, with 80% power at alpha<0.05, we can detect a fold change of 1.4 for microbiome differences and a fold change of 1.6 for mRNA transcripts. Power calculations were performed with the RNAseqPower package in R. For microbiome data we calculated a biological coefficient of variation of 0.3, and for RNAseq, we used a coefficient of variation of 0.4. We estimated the expected minimum fold change that we could observe for each group based on the sample size, sequencing depth, and a significance level of 0.05 0.05.

Data exclusions

No data were excluded from this analysis.

Replication

The combination of three unique and independent datasets in this study allows us to answer specific questions in a replicatable way.

Randomization

Subjects were randomized in the treatment cohort according to the methodology in Walsh et al. 2020.

Blinding

This manuscript is not evaluating a primary endpoint in a trial. We were all unblinded to treatment allocation.

Reporting for specific materials, systems and methods

We require information from authors about some types of materials, experimental systems and methods used in many studies. Here, indicate whether each material, system or method listed is relevant to your study. If you are not sure if a list item applies to your research, read the appropriate section before selecting a response.

Materials & experimental systems

n/a	Involved in the study
☒	☐ Antibodies
☒	☐ Eukaryotic cell lines
☒	☐ Palaeontology and archaeology
☒	☐ Animals and other organisms
☐	☒ Human research participants
☐	☒ Clinical data
☒	☐ Dual use research of concern

Methods

n/a	Involved in the study
☒	☐ ChIP-seq
☒	☐ Flow cytometry
☒	☐ MRI-based neuroimaging

Human research participants

Policy information about studies involving human research participants

Population characteristics	Longitudinal arms of the study: Donors were enrolled through the Clinical Trials Unit at GHESKIO. Pulmonary TB was diagnosed by clinical symptoms, chest radiograph consistent with pulmonary TB, and positive molecular testing. All participant samples were deidentified on site using a barcode system before they were shipped to Weill Cornell Medicine (WCM)/Memorial Sloan Kettering Cancer Center (MSKCC) for analysis. All clinical metadata was collected on site and managed through the REDCap data management system. Study population consisted of men and women ages 18 - 65, diagnosed with pulmonary tuberculosis via: sputum-microscopy smear-positive (2+ or 3+) within 14 days plus Sputum GeneXpert positive within 14 days plus Chest radiograph consistent with M. tuberculosis within 14 days. TB treatment naïve at time of enrollment. Bodyweight > 40kg. Negative HIV test within 30 days. Able to complete activities of daily living (ADLs). All participants had to agree not to participate in a conception process (i.e. active attempt to become pregnant or to impregnate, donate sperm, in vitro fertilization)<br>All female participants had to agree to use barrier methods such as condoms as well as hormonal contraception for dual prophylaxis. Able to give informed consent and demonstrate understanding of this study and willingness to participate in this study. Willing to be hospitalized for 2 weeks. All additional information related to the clinical trials are publicly available under https://clinicaltrials.gov/ct2/show/study/NCT02684240.<br><br>Cross-sectional validation arm: For family contacts we recruited families of active pulmonary TB patients where at least two siblings within the family were diagnosed with active TB. These criteria were designed to select for households with high risk of transmission of Mtb. Household contacts were then recruited if they had been sleeping in the same house with a TB case for at least one month during the six months prior to the TB case diagnosis. Contacts underwent clinical screening for active TB symptoms and IGRA testing.<br>Healthy donors without history of TB contacts or disease were recruited from the same community as a control group for exposure and also underwent clinical screening for active TB symptoms and IGRA testing. All donors provided informed consent prior to peripheral blood donation for whole blood collection for RNAseq and stool submission for DNA extraction and 16S rDNA sequencing.<br><br>Significant results (FDR<0.05, FC>1.5) in our differential gene expression analyses were significant with or without the inclusion of covariates (y ~ 1 + (1|subject) vs y~ 1+ age + batch + (1|subject) produce similar numbers of differentially expressed genes).
Recruitment	Recruitment was in Port au Prince, Haiti through the GHESKIO hospital system, and funded through our NIH TBRU grant. Also, see above (Population Characteristics).
Ethics oversight	All volunteers provided written informed consent to participate in this study. All human studies were reviewed and approved by the IRBs of both Weill Cornell Medicine and Groupe Haitien d'etude du Sarcome de Kaposi et des Infections Opportunistes (GHESKIO) Centers (Port-au-Prince, Haiti). Participants provided informed consent prior to peripheral blood draw for whole blood collection and stool collection for 16S rDNA sequencing. All methods and procedures were performed in accordance with the relevant institutional guidelines and regulations.

Note that full information on the approval of the study protocol must also be provided in the manuscript.

Clinical data

Policy information about clinical studies

All manuscripts should comply with the ICMJEguidelines for publication of clinical research and a completedCONSORT checklist must be included with all submissions.

Clinical trial registration	NCT02684240

Study protocol	The protocol is attached, however this is not the primary clinical trial manuscript.
Data collection	Subjects were recruited and samples collected from 2016 - 2019.
Outcomes	N/A
